# Analysis of Successful Behaviors Leading to Groundwork Scoring Skills in Elite Judo Athletes

**DOI:** 10.3390/ijerph19063165

**Published:** 2022-03-08

**Authors:** Xurxo Dopico-Calvo, Eliseo Iglesias-Soler, Luis Santos, Eduardo Carballeira, Xián Mayo

**Affiliations:** 1 Performance and Health Group, Department of Physical Education and Sport, University of A Coruna, 15179 A Coruña, Spain; xurxo.dopico@udc.es (X.D.-C.); eliseo.iglesias.soler@udc.es (E.I.-S.); lsanr@unileon.es (L.S.); 2 Department of Physical Education and Sport, University of León, 24071 León, Spain; 3 Observatory of Healthy and Active Living of Spain Active Foundation, Centre for Sport Studies, King Juan Carlos University, 28942 Madrid, Spain; xian.mayo@urjc.es

**Keywords:** key performance indicator, lateral structure, grip, grappling, ne-waza

## Abstract

The present study aimed (1) to propose an approach of observational analysis of the preceding standing judo (tachi-waza (TW)) context to a groundwork (ne-waza (NW)) grappling score (NWGS), and (2) to analyze the outcomes of applying such a model in high-level judoists. We conducted an observational analysis of 176 NW scoring actions of 794 combats observed in Baku’s World Judo Championships of 2018. Women scored more NWGS, performing more corporal controls but less segmental controls compared with the men. Moreover, NWGS were scored predominately during the second and third minutes of combat, independently of the sex or the weight category. Most NWGS occurred after an asymmetrical lateral structure, without showing associations with a particular type of NWGS. The movement structure of the attacking action during TW leading to an NWGS was predominantly techniques without turn, followed closely by techniques with turn, and barely performed after supine position techniques. Data showed that NWGS occurred more frequently after a failed TW attack (68.6%) than after a scored TW attack (31.4%). The TW attacker achieved NWGS with a higher frequency (62%) than the TW defender (38%), who mainly took advantage of a failed TW attack (98.5% vs. 1.5%, after failed vs. scored TW, respectively). The grip configurations most frequently employed during TW were dorsal-sleeve and flap-sleeve; overall, frontal grips were predominant over dorsal grips. However, no specific TW grip was related to success or grip progression before an NWGS. Our results will help judo coaches understand the influence of these factors on judo performance and optimize the planning and execution of technical–tactical content.

## 1. Introduction

Judo is an Olympic and Paralympic high-intensity intermittent combat sport. Many physical attributes combined with optimal technical–tactical development become necessary in order to achieve competitive success [1,2,3]. Judoists must learn skills with high coordinative demands and make multiple decisions quickly to unbalance and throw their opponents in standing judo [4]. Furthermore, a judoist can choose to fight on the ground (i.e., ne-waza (NW)) when at least one throws, pulls to the ground, or blocks the other, causing the opponent to touch with his/her trunk (without achieving maximal score) or his/her knees and upper limbs simultaneously ([5], art. 10). At that moment, the objective of both judoists is to hold the opponent down using a grappling skill such as an immobilization (i.e., osaekomi-waza (OW)) or getting submission (through a neck choke-holding, i.e., shime-waza (SW), or locking the elbow, i.e., kansetsu-waza (KW)). In international competitions, judo medalists usually fight between five and seven bouts lasting up to four regulatory minutes ([5], art. 6) If they reach the end of the bout even on the scoreboard, the regulatory time is extended, fighting in the golden score (GS) time. The first judoka scoring during the GS wins, whether throwing, getting an immobilization, submitting in groundwork, or causing an accumulation of three sanctions onto the opponent ([5], art. 13.4).

Researchers have identified several phases within the effort period of the judo bout: approach, gripping, standing judo (i.e., tachi-waza (TW)), and NW [3,6]. To date, research in NW has mainly examined time-motion indicators [7,8,9], such as the time spent fighting [9], along with the types of techniques used and the efficiency of these techniques [10]. Observational studies have found that the NW duration varies from 6.9 s to 17.4 s, depending on whether the score is achieved or not, respectively, expending from 10 to 30% of the effort time fighting on the ground [11]. While this is relatively stable despite the judo rules changes through practically two decades, it has been reported that NW sequences are longer in the absolute age category than in the younger one [7].

During judo combats, referees pay attention and evaluate the dynamic behavior of the judoists when they are fighting on the ground, stopping the fight if there is no evident progress ([5], appendix D, art. 11). As NW is the consequence of what happened previously on TW, it seems essential to analyze this passage from the preceding TW context to an NW grappling skill scoring (NWGS), also considering the NWGS type (OW, SW, or KW). To the best of our knowledge, only one peer-review article has systematically studied some determinants of the NWGS and the transition from standing judo [12]. That study analyzed the preceding TW context to an NWGS during the senior and junior Judo World Championships. They studied the association between the scoreboard at the transition to NW, the transition rhythm attending to the time passed until NWGS, and the position of the judoist receiving the NWGS during the transition. Nevertheless, the TW particulars such as the lateral structure (i.e., symmetry or asymmetry) and grips adopted during TW were not analyzed. Nevertheless, it is well known that asymmetrical lateral structure and movement structure of the techniques showing turns presented a higher preponderance of scorings during TW, potentially also influencing scoring during NW [13]. This information would be very valuable to understand the determinants of NWGS and to optimize the training processes and the rivals’ scouting. Therefore, we established two objectives: (1) to propose a model of observational analysis of the preceding TW context to an NWGS, and (2) to analyze the outcomes of applying such a model in high-level judoists.

## 2. Materials and Methods

### 2.1. Design

The present study assessed all the critical data of the preceding TW context to an NWGS during the Senior 2018 Judo World Championships held in Baku (Azerbaijan). Data were collected using the program Lince PLUS version 1.3.2. This study used an ad-hoc instrument specifically designed to observe and collect the information in a database in order to subsequently carry out the statistical analysis. We performed a cross-sectional analysis based on the observation of complete judo combats extracted from an open-access website (https://www.ijf.org/competition/1591/contests accessed on 20 January 2020) in secondary form and not generated by experimentation. Therefore, there were no ethical issues in examining or interpreting them. In addition, the personal identification of the judoists was coded for treatment and data analysis.

The championship analyzed involved a total of 14 categories, structured by gender and weight (women: −48 kg, −52 kg, −57 kg, −63 kg, −70 kg, −78 kg, and +78 kg; men: −60 kg, −66 kg, −73 kg, −81 kg, −90 kg, −100 kg, and +100 kg). Thus, 794 combats and 176 NWGS were analyzed (eight actions were excluded due to the inability to categorize them). A total of 755 athletes (297 women and 458 men) from 124 nations participated in the matches evaluated.

### 2.2. Procedures

Briefly, since 2018, there have been two scoring possibilities during judo combat—ippon and waza-ari [14]. When the first ippon is scored, the combat is finished. Two waza-ari are equivalent to an ippon, and thus the combat is also finished. The athlete who scored an ippon or two waza-ari wins, and if an ippon or two waza-ari were not scored, then the athlete with the higher punctuation (i.e., waza-ari) wins. While considering NWGS, there are two possible situations: (a) no scoring during TW but scoring at least a waza-ari (or an ippon) on NW, or (b) one of the two athletes scores a waza-ari during TW, and afterward, there is another scoring action during NW, from the same athlete or not. Another possibility is after not having a TW scoring, having two following NWGS in the same phase in which the first one is an immobilization scoring waza-ari, but the chances of this are unlikely.

We collected several variables to determine the preceding TW context features to an NWGS and the relevant information of the NWGS itself. Regarding NWGS, we considered the temporal units, sequences duration, weight categories, and sex. Concerning the temporal units, the minute of the NWGS was collected considering five-time units (TU): 0 to 59 s (TU1), 60 to 119 s (TU2), 120 to 179 s (TU3), 180 to 240 s (TU4), and golden score time (GS). The 17 TUs corresponding to GS were analyzed as a whole, that is, as extra or additional time. The length of GS ranged from 34 s and 312 s, with a mean of 144 s. In addition, we registered the type of NWGS (i.e., OW, SW, or KW) and the number of position progressions, explained as a change in the posture of the NW-attacker or NW-defender, and structured from 0 to 6. To simplify the analysis, we recoded the position progressions in short position progression ((SPP) from none to one posture change) and long position progression ((LPP) from two to six posture changes). The total number of the type of NWGS (i.e., OW, SW, or KW) was analyzed considering the weight category, pooled as light (−60 kg and −66 kg in men, and −48 kg and −52 kg in women), medium (−73 kg, −81 kg, and −90 kg in men, and −57 kg, −63 kg, and −70 kg in women), and heavy (−100 kg and +100 kg in men, and −78 kg and +78 kg in women), and the gender of the judoists.

Regarding the lateral structure of the preceding TW context, the shoulder or hip turning behavior of the one breaking guard distance (i.e., the attacker) and the stance position of who received the action (i.e., the defender) were considered. For an attacker, right or left turning behavior was considered based on if the rotation of the right shoulder turned to the left (anticlockwise) or the left shoulder turned to the right (clockwise), or if a right or left dynamic leg applied a technique, respectively. A defender was identified as right or left positioned when the advanced leg was the right or left, respectively. Thus, the lateral structure of the TW action could be symmetrical when the attacker and the defender were right versus right or left versus left, or asymmetrical, when they adopted different relative positions, right versus left or left versus right. Detailed explanations of the topic can be found elsewhere [13,15].

We organized the movement structure of the attacking action during TW following the motor criteria described in previous work of our group [15]. Briefly, this movement structure criterium classifies the techniques according to the occurrence of turning before the TW action (T-TW), techniques without turning before the TW action (WT-TW), or techniques that are performed during supine position (SP-TW). Furthermore, we registered the success or failure of the TW action before NW and the fact that the TW performer scored or was scored in NW.

Regarding the attacker grips during the preceding TW context to an NWGS, two analyses were implemented. Firstly, considering all the gripping possibilities: dorsal-flap, dorsal-sleeve, dorsal-free, flap-flap, flap-sleeve, flap-free, sleeve-sleeve, and sleeve-free. Secondly, considering the hand’s position regarding the opponent body, in which one hand was dorsal (dorsal-flap, dorsal-sleeve, dorsal-free) or both were frontal (flap-flap, flap-sleeve, flap-free, sleeve-sleeve, and sleeve-free). The latter type of analysis was considered for studying the grip progression in NW, i.e., those actions in which a control grip was observed (when at least one hand was maintained from the TW attack to NWGS) or when there was an ongoing grip (both hands showing new grips from the TW attack to the NWGS). A description of the observational instruments of the study is shown in Table 1.

### 2.3. Statistical Analysis

Descriptive data are presented as percentages. Most of the analyzed variables were categorical, and only one variable, length of sequences, was quantitative. Therefore, the length of sequences, in raw values and after logarithmic transformation, was tested for normality by the Shapiro–Wilk test. As normality assumption was violated with and without transformation, we applied a non-parametric procedure for categorical and quantitative variables. A one-sample Pearson’s chi-squared (χ^2^) test was implemented to analyze the hypothesis of a uniform distribution and to evaluate the over or under-representation of the different categories within each variable. Pearson’s chi-squared (χ^2^) was implemented to analyze the association between the variables. When a significant association was detected, its interpretation was carried out considering both standardized residuals (residuals with absolute values greater than two were deemed significant) and performing a correspondence analysis. When significant, the strength of associations was reported as Cramer’s V (V). A Cramer’s V value lower than 0.20 indicated a small effect, a value within the range of 0.21–0.35 indicated a medium effect, and a value larger than 0.35 indicated a larger effect [16]. For contrasting the length of the sequences between the type of NWGS (i.e., OW, SW, and KW), a Kruskal–Wallis one-way analysis of variance with post-hoc paired comparisons by a Mann–Whitney U test with Bonferroni adjustment was performed. The effect size for these differences was reported by the rank biserial correlation (r) [17]. We conducted an inter and intra-observer consistency analysis for the grip progression variable. In the first case, we selected an automatic random sample of around 25% of the actions, and two independent observers (X.D.C. and E.C.) classified the grip progression. After that, we calculate the kappa statistic, obtaining a high level of agreement (κ = 0.876; *p* < 0.00). We accomplished an intra-rater reliability analysis after a new random sample of around 25% of the actions. After that, the primary observer (X.D.C.) classified the grip progression twice in sessions 72 h apart. The results were analyzed by McNemar’s test, which reflected no changes in the classification of the actions (1 change out of 36 actions; *p* = 1.000). All the statistical analyses were executed with SPSS 27 (IBM, Chicago, IL, USA). The level of significance was set at 0.05.

## 3. Results

### 3.1. Temporal Units, Sequences Duration, Weight Categories, and Sex

Most of the NWGS occurred within TU2 (27.8%) and TU3 (32.4%), whereas the scores obtained during GS were the least frequent (9.7%). One sample Chi-square test rejected the uniform distribution of cases between TU (*p* < 0.001), and the residuals concerning the expected frequency were −14.2, 13.8, 21.8, −3.2, and −18.2 for TU1, TU2, TU3, TU4, and GS, respectively. The NW sequences duration of OW scoring action (27 ± 16 s) were higher (*p* < 0.001) compared to KW (17 ± 18 s) and SW (16 ± 16 s). The univariate analysis showed a similar distribution of NWGS between weight categories (39.2%, 33.5%, and 27.3% for light, medium, and heavyweight, respectively; *p* = 0.152). On the other hand, the women achieved more NWGS than the men (58.5% and 41.5%, respectively; *p* = 0.024). The more predominant type of NWGS was the OW (74.4%), followed by SW (14.2%) and KW (11.4%), being the distribution non-uniform between categories (*p* < 0.001; residual: 72.3, −38.7 and −33.7 for OW, SW, and KW, respectively). The type of NWGS was not significantly associated with weight categories (*p* = 0.170), but instead with sex (*p* = 0.035; V = 0.195). Correspondence analysis showed negative scores for women and OW (dimension score = −0.372 and −0.257 respectively), and positive scores for men and KW and SW (dimension score = 0.525, 0.851, and 0.668 respectively).

### 3.2. Position Progressions and the Type of NW Grappling Skill Scoring Action

The number of position progressions ranged from 0 to 6, with a progressive decrease in their frequencies as follows: 31.3%, 29.5%, 21.6%, 8.5%, 5.7%, 2.3%, and 1.1% from 0 to 6 stages, respectively. One sample Chi-square test rejected the uniform distribution between categories (*p* < 0.001), with greater residuals observed for levels 0 and 1 (29.9 and 26.9, respectively). While recoding the position progressions, SPP (0–1 position change) and LPP (2 or more position changes) were associated with the type of NWGS (i.e., OW, SW, or KW) (*p* = 0.003; V = 0.253). Correspondence analysis showed proximity for KW and LPP (−0.642 and −0.627), and OW and SPP (0.290 and 0.405, respectively). Furthermore, the dimension score for SW was closer to LPP (−1.008).

### 3.3. Lateral Structure, Movement Structure, Scoring in TW, and Relationship between Scoring in TW vs. Scoring in NW

**Figure 1:**
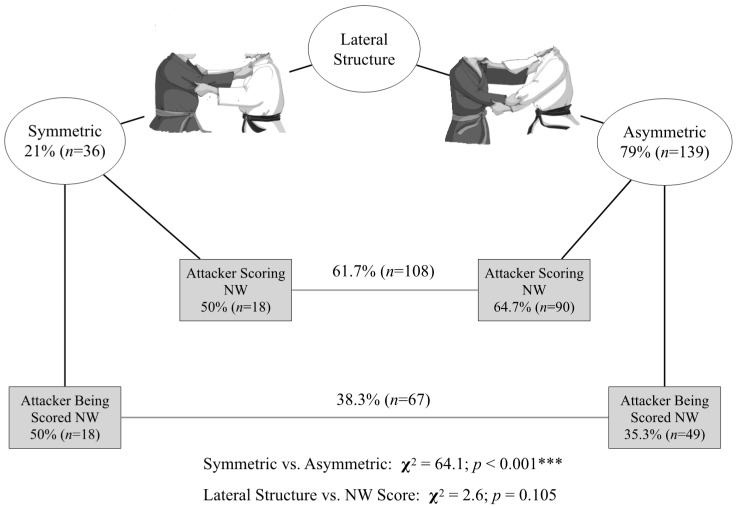
Lateral structure vs. success in ne-waza (NW) by the attacker. Significant differences: *** *p* < 0.001.

Considering the judoists’ TW lateral structure adopted before an NWGS, we observed that asymmetric positions predominated compared with symmetric positions (79% of cases; *p* < 0.001). Nevertheless, the lateral structure was not associated with scoring during NW by the TW attacker (Figure 1).

**Figure 2:**
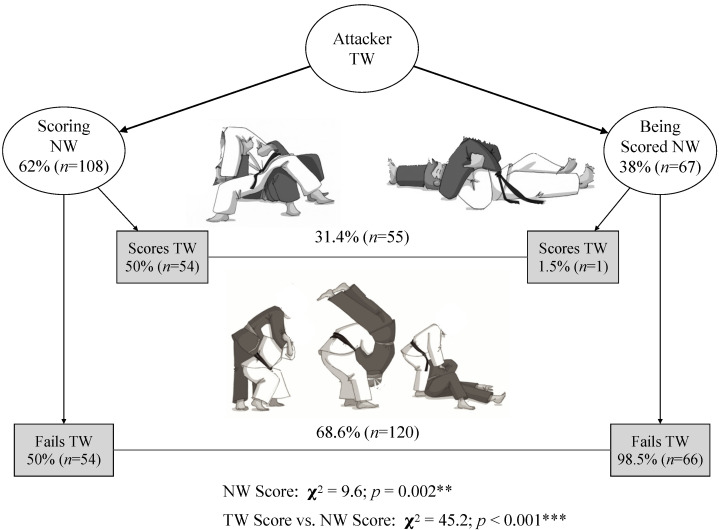
Attacking role in tachi-waza (TW) and the relationship between scoring in ne-waza (NW) and scoring in TW. Significant differences: ** *p* < 0.01; *** *p* < 0.001.

There was no uniformity in the type of movement structure distribution during a TW attacking action before an NWGS (*p* < 0.001). The most frequent types of movement structure used were WT-TW (47.2%) and T-TW (40.9%), and the least frequent was SP-TW (11.9%), whereas the residuals for the movement structure types were 24.3, 13.3, and −37.7, respectively. Furthermore, a significant association was observed between scoring during TW and scoring during NW (*p* < 0.001; V = 0.508). The one attacking during TW scored 62% of the time during NW (scoring vs. being scored in NW, *p* = 0.002) (Figure 2), even considering that he/she did not score half of the TW attacks (54 out 108). On the other hand, the one attacking during TW received an NWGS 38% of the time, considering that 98.5% of these losses came after not scoring during a TW action and only 1.5% after scoring during TW (Figure 2). NWGS were preceded predominantly by unsuccessful TW actions (68.6%; *p* < 0.001; Figure 2). Consequently, the correspondence analysis showed positive values for an unsuccessful attack during the TW (dimension score = 0.482) ending losing during NW (dimension score = 0.905). In contrast, the TW scoring action ending scoring during NW obtained negative scores (−1.053 and −0.561, respectively).

### 3.4. Attacking Grips during TW Context and Grips Progression during NW Scoring Actions

**Figure 3:**
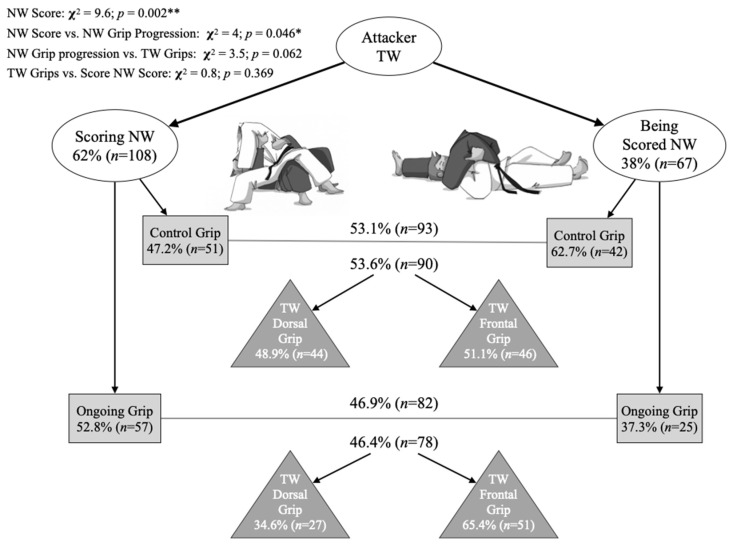
Associations between ne-waza (NW) score (circles), grip progression during NW (squares), and attacking grips during the preceding tachi-waza (TW) (triangles). Grip progression was categorized as control grip (when at least one hand was maintained from the TW attack to NW scoring action) and ongoing grip (both hands showed new grips from the TW attack to the NW scoring actions). Percentages above the grey lines represent the distribution of grip progression categories. Percentages below the line represent the distribution of grip progression categories when crossed with TW grips. Significant differences: * *p* < 0.05; ** *p* < 0.01.

The most frequent attacking grips during the preceding TW context to an NWGS were as follows: dorsal-sleeve (28.4%), flap-sleeve (19.9%), flap-free (11.4%), sleeve-sleeve (10.2%), dorsal-flap (9.1%), sleeve-free (8.5%), flap-flap (5.1%), and dorsal-free (2.8%). One sample Chi-square test rejected the uniform distribution between categories (*p* < 0.001), with an overrepresentation observed for dorsal-sleeve and flap-sleeve. When we grouped the attacking grips during the TW context preceding an NWGS into dorsal and frontal, the latter was predominant (57.7%; *p* = 0.045). Meanwhile, grip progression for an NWGS presented a balanced distribution between control grip (52.8%, *n* = 93) and ongoing grip (47.2%, *n* = 83) categories (*p* = 0.451). Grip progression categories during NW were significantly associated with NWGS achieved or received by the one attacking during TW (*p* = 0.046; V = 0.151) (Figure 3). The correspondence analysis suggested proximity between categories of TW attacker scoring during NW and ongoing grip (dimension score was −0.306 and −0.413, respectively) and between TW attacker being scored during NW and control grip (dimension scores were 0.493 and 0.364, respectively). On the other hand, attacker grips during the preceding TW context were associated neither with grip progression nor with NWGS by attacker categories (*p* = 0.062 and *p* = 0.369, respectively) (Figure 3).

## 4. Discussion

The main objectives of the present study were to propose a model of observational analysis of the preceding TW context to an NWGS and to analyze the outcomes of applying such a model in high-level judoists. For that purpose, we analyzed 176 NW scoring actions of the World Judo Championships of 2018 in Baku.

NWGS were scored predominately within TU2 and TU3. This result partially agrees with what was observed in the World Championship of 2017 [12], where the authors found the highest occurrence from TU2 onwards, without a decrease of frequency from TU3. Based on these results, it seems unlikely to get NWGS in the first minute of the match. Moreover, we observed a longer duration of the NW sequence depending on the NWGS type employed, with OW sequences (∼27 s) being around 10 s longer than KW (∼17 s) and SW (∼16 s). Some authors have observed NW sequences duration ranging from 4 to 19 s with high deviation from the mean values for an absolute age category (male and female), like what was presented in our study [3,7,11,18]. However, previous studies did not present the duration per NWGS type, making comparisons difficult. Knowing the time invested to be successful in a determined NWGS type could have crucial strategic value when approaching the final stages of matches.

We did not find differences between weight categories in the distribution of NWGS or the NWGS type. Two studies in the literature have observed differences in the frequency and duration of NW periods between weight categories. Soriano et al. [19] found that lightweight and middleweight categories spent more time in NW. Conversely, Soto et al. [20] found that middleweight and heavyweight categories demonstrated a higher NW frequency than lightweight categories. However, these authors analyzed the frequency and duration of NW periods without considering if a score was achieved or not, thus making the comparison difficult.

On the other hand, we observed more NWGS in women than in men (58.5%), and sex was associated with the type of NWGS used. Indeed, women performed more OW, and men performed more KW and SW. Our results are in contrast with the similar distribution of techniques executed to score in NW by women and men during the World Judo Championship of 2017 [12]. Notwithstanding, and according to our results, a study analyzing 1311 judo bouts from 36 different high-level judo competitions (Olympic Games, World Masters, World and Continental Championships, and World Cups) found that women performed a higher frequency of OW [21].

It has been reported that laterality in TW positions (i.e., having a right or left stance) influences the NW phase [22]. However, the observation of rotation directions in the execution of standing skills, without considering the lateral structure, does not provide enough information to understand the imbalance opportunities that judoists might cause to throw the opponent. As far as we know, no studies have analyzed the association of the lateral structure of TW actions with NWGS. Our results show that asymmetrical positions predominated (79%) over symmetrical positions, but there was no association with a particular type of NWGS. In line with this, asymmetrical and symmetrical positions influence standing judo opportunities [13]. Therefore, more studies are warranted to analyze successful and unsuccessful NW periods so as to bring valuable information about the efficiency of making NW transitions from a particular TW lateral structure.

We also observed that the movement structure of the attacking action during TW leading to a score during NW was predominantly WT-TW (47.2%) and T-TW (40.9%), with SP-TW (11.9%) being executed less frequently before an NWGS. According to our results, an NWGS occurred more frequently after a failed TW attack (68.6% of the time), and to score during NW it is better to be the attacker (62% of scored NW) and get a score during a TW attack, as indicated by the correspondence analysis. If the judoist is the TW defender, they can get success in NW (38% of the scored NW) only taking advantage of a failed throw attempt (98.5% of the time). These results are like those obtained in the 2017 World Judo Championships, where the authors observed 67.3% scored in NW by the TW attacker [12].

The present study is the first work in the literature that has considered grips during TW with NWGS. Frontal TW grips were predominant in NW, and the grip configurations more frequently employed during TW were the classical ones, dorsal-sleeve, and the flap-sleeve. However, no specific TW grip was related to success or grip progression on the ground. Future studies designs should determine if some grips are more efficient than others to score on the ground by analyzing successful or unsuccessful grappling techniques. Furthermore, sports scientists should clarify if the gripping efficiency to obtain a score in NW is more related to the chances of a specific grip configuration to apply grappling techniques or to the volume of the practice of the classical grips linking with NW work. Even though grip progression (control grip or ongoing grip) presented a balanced distribution in NWGS, ongoing grips were employed with a higher frequency to win on the ground by the TW attacker, and the control grip was used more to score by the TW defender.

Positions progression on the ground can give important information for judoists to structure a tactical plan based on the quantity of work applied to get a score for a grappling technique. Short position progression (from 0 to 1 position change) was associated with OW, and long position progression was associated with KW and SW. Judoists may choose to search for KW and SW when do not they fatigued and have an advantage on the scoreboard. Therefore, more position changes should avoid being stopped by the referee, as the regulatory judo rules establish that the fight must be stopped in case of no evident progress (art. 11-2e, [5]). Moreover, we observed that the NWGS more predominant was the OW (74.4%), followed by SW (14.2%) and KW (11.4%), agreeing with the grappling skills distribution previously observed in high-level judo players [12,23]. A potential limitation of this study might be that only NWGS were analyzed, and therefore it was not possible to estimate the likelihood of a successful action during NW. Further studies are needed to expand our analysis to larger samples so as to confirm the outcomes observed in this work.

## 5. Conclusions

The analysis of the key performance indicators of the preceding TW context to NWGS in elite judoists showed that women scored more NWGS, performing in proportion more OW but less KW and SW than men. Moreover, NWGS were scored predominately during the second and third minutes of combat, independently of the sex or the weight category. Most NWGS occurred after an asymmetrical lateral structure, without showing associations with a particular type of NWGS. The movement structure of the attacking action during TW leading to a score during NW was predominantly techniques without turn, followed closely by techniques with turn and almost anecdotal techniques performed during the supine position. Data showed that NWGS occurred more frequently after a failed TW attack (68.6%) than after a scored TW attack (31.4%), but that the TW attacker scores with a higher frequency. The TW attacker achieved NWGS with a higher frequency (62%) than the TW defender (38%) who mainly took advantage of a failed TW attack (98.5% vs. 1.5%, after failed vs. scored TW). A judoist defending an attack during TW was less prone to score during NW, only taking advantage of a failed throw attempt. The most frequently employed grip configurations during TW were dorsal-sleeve and flap-sleeve, but overall, frontal grips were predominant over dorsal grips. However, it is essential to note that no specific TW grip was related to success or any level of grip progression before an NWGS.

In summary, our results indicate that judo coaches and athletes seeking to increase the probability of scoring in NW should introduce the following into their training sessions:

- Practice the connection of all grappling skills in NW after failed attacks preceded by TW situations with asymmetric lateral structure and when the attacker uses skills without turn.
- Use preferably dorsal-sleeve and flap-sleeve grips in the preceding TW actions.
- Train both roles, attacker and defender.

## Figures and Tables

**Table 1:** Observational instrument of the study.

Concept	Abbreviations	Description
**Temporal Units**	TU	
	TU1	1st temporal unit (from 0 to 59 s)
	TU2	2nd temporal unit (from 60 to 119 s)
	TU3	3rd temporal unit (from 120 to 179 s)
	TU4	4th temporal unit (from 180 to 240 s)
	GS	Golden score
**Tachi-waza**	TW	Standing Judo
**Ne-waza**	NW	Groundwork Judo
**Ne-waza Grappling Score**	NWGS	Groundwork grappling skills for scoring in judo: osae-komi-waza, kansetsu-waza, and shime-waza
Osae-komi-waza	OW	Immobilization techniques
Kansetsu-waza	KW	Neck choke-holding techniques
Shime-waza	SW	Elbow locking techniques
**Ne-waza Sequence Duration**		OW sequence duration comprised from NW beginning to the referee indication of the beginning of immobilization. Time spent to get a score once immobilization started was not computed. KW and SW sequence duration comprised from NW beginning up to score by adversary surrender
**Ne-waza Grip Progression**		
Control grip		The judoka who scored in NW maintained at least one hand from the TW attack to NWGS
Ongoing grip		The judoka who scored in NW changed both hands (i.e., new grips) from the TW attack to NWGS
**Ne-waza Position Progression**		
	SPP	Short position progression consists of zero or one change in the posture of the NW-attacker or NW-defender
	LPP	Long position progression consists of at least two to six changes in the posture of the NW-attacker or NW-defender
**Tachi-waza Attacker Grips**		
Dorsal		At least one hand is in the dorsal part of the adversary judo suit. For example: dorsal-flap, dorsal-sleeve, and dorsal-free
Frontal		Both hands were in the frontal part of the adversary judo suit. For example, flap-flap, flap-sleeve, flap-free, sleeve-sleeve, and sleeve-free
**Tachi-waza Lateral Behavior**		
Attacker right turning behavior		If the rotation of the right shoulder turns to the left (anticlockwise), or a dynamic right leg applies a technique
Attacker left-turning behavior		If the rotation of the left shoulder turns to the right (clockwise), or a left dynamic leg applies a technique
Defender right behavior		If the advanced leg was the right at the moment of the attack
Defender left behavior		If the advanced leg was the left at the moment of the attack
**Tachi-waza Lateral Structure**		
Symmetrical		The attacker and the defender were right versus right or left versus left
Asymmetrical		The attacker and the defender adopted different relative positions, right versus left or left versus right
**Tachi-waza Movement Structure**		
	T-TW	Tachi-waza techniques with turning
	WT-TW	Tachi-waza techniques without turning
	SP	Tachi-waza techniques performed during supine position

## Data Availability

The data presented in this study are available on request from the corresponding author.
